# A New ‘*cyclotis*-morphotype’ Species of Tube-Nosed Bat (Chiroptera: Vespertilionidae: *Murina*) from China

**DOI:** 10.3390/ani15010075

**Published:** 2025-01-01

**Authors:** Xin Mou, Yishun Qian, Wen Wang, Wenxiang Zhang, Jianjie Wang, Song Li

**Affiliations:** 1 Kunming Natural History Museum of Zoology, Kunming Institute of Zoology, Chinese Academy of Sciences, Kunming 650223, China; mouxin@mail.kiz.ac.cn (X.M.);; 2 Yunnan Huanglianshan National Nature Reserve Management Bureau, Lvchun 662599, China; 3 Yunnan Key Laboratory of Biodiversity Information, Kunming Institute of Zoology, Chinese Academy of Sciences, Kunming 650201, China

**Keywords:** *Murina*, new species, taxonomy, morphology, China, Cyt *b*

## Abstract

During an examination of various specimens previously collected from different locations at different times, we discovered four *Murina* specimens that had been collected in October and December 2023 from the Huanglianshan National Nature Reserve, Lvchun, Yunnan, China. Morphologically, these specimens can be distinguished from *M. pluvialis* and other congeneric species based on a combination of body size, hair distribution, fur colour, and skull and teeth characteristics; molecularly, an analysis of Cyt *b* and COI gene sequences showed that these specimens form a monophyletic group with *M. pluvialis* with high posterior probability and bootstrap support values. Furthermore, the genetic distance between our specimens and *M. pluvialis* was greater than the minimum threshold for interspecific differentiation, indicating that they are phylogenetically close but have diverged. Based on the above assessment of morphological characteristics and molecular data analysis, these specimens were determined to represent a previously unidentified species, designated *Murina lvchun* Xin Mou & Song Li, sp. nov.

## Simple Summary

In this paper, a new *Murina* species is described from Lvchun, Yunnan, China, based on morphological and molecular evidence. Morphologically, the new species is most similar to *Murina pluvialis* but can be distinguished from this and all other congeners by a combination of morphological characteristics. Genetically, the new species is most closely related to *M. pluvialis* based on the phylogenetic tree of the mitochondrial cytochrome b (Cyt *b*) and cytochrome oxidase subunit I (COI) gene sequences, but there is still a genetic distance of 7.2–7.4%. The discovery of the new species not only enriches our understanding of biodiversity but also highlights the importance of southwest China as a biodiversity hotspot.

## 1. Introduction

The family, Vespertilionidae Gray, 1821 [1] is the largest family in the order Chiroptera and the second largest mammalian family after Muridae [2]. According to Simmons [3], this family has 407 known species, 48 genera and 6 subfamilies (Vespertilioninae, Antrozoinae, Myotinae, Miniopterinae, Murininae, and Kerivoulinae) as of 2005. Over the last two decades, a large number of new species in this family have been discovered. As of 13 July 2024, The Mammal Diversity Database of the American Society of Mammalogists (ASM) has recorded 531 species in 60 genera [4]. As the family Vespertilionidae is the most widespread family in the order Chiroptera (found on all continents except Antarctica) [5] and as bats are capable of flight and are usually nocturnal, it is not difficult to speculate that there are still many unreported species of the family Vespertilionidae in remote areas.

Miller [6] named the subfamily Murininæ—including only *Murina* and *Harpiocephalus*—based on the following characteristics: anterior upper premolars (P^2^) slightly reduced, slightly smaller than the posterior upper premolars, and essentially similar in morphology; molars essentially normal or considerably modified, with the metaconid clearly the largest cusp of the first (M^1^) and second molars (M^2^); and the nostrils clearly protruding in a tubular shape. The above taxonomic hypothesis is generally accepted [1,7,8,9,10]. The genus *Harpiola* was originally introduced as an independent genus by Thomas [11], with *Murina grisea* as its type species. Bhattacharyya [12] provided a detailed description of the second *grisea* specimen and conducted a comprehensive re-evaluation of *Harpiola*. In describing a new species (*Harpiola isodon*), Kuo et al. [13] presented a systematic account of the diagnostic characteristics distinguishing between *Harpiola* and *Murina*. Both authors upheld the validity of the genus *Harpiola*. Kruskop et al. [14] and Li et al. [15] have reported on specimens of *H. isodon* from Vietnam and mainland China, respectively. The validity of the genus *Harpiola* is generally accepted.

The genus *Murina* Gray, 1842 [16] is the largest genus in the subfamily Murininae. Simmons [3] confirmed the presence of 17 species within *Murina* in 2005, and its membership has more than doubled over the last two decades, with a large number of new species having been reported [17,18,19,20,21,22,23,24,25,26,27,28,29,30,31,32,33]. At present, 41 species of the genus *Murina* are listed in the Global Biodiversity Information Facility (GBIF) [34] and 39 species are recognised by the Mammal Diversity Database of ASM [4], with 21 species distributed in China according to the Catalogue of Mammals in China [35]. The body size of *Murina* is usually significantly smaller than that of *Harpiocephalus* (except for a slight overlap of *Murina leucogaster* with *Harpiocephalus* in forearm length), and *Murina* can be distinguished from *Harpiocephalus* based on its skull and dentition [36,37]. It also can be distinguished from *Harpiola* according to a number of diagnostic dental characteristics [13]. Corbet and Hill [7] divided *Murina* into two groups: the ‘*suilla*-group’ and the ‘*cyclotis*-group’, based on the relative size of the upper canines (C^1^), the first (P^2^) and second upper premolars (P^4^), and the position of the incisors. This classification remains in application to species described subsequently [7,9,17,18,19,20,21,22,24,25,26,33,36]. However, the two morphogroups do not represent distinct phylogenetic lineages and will, therefore, be referred to hereafter as the ‘*suilla*-morphotype’ and the ‘*cyclotis*-morphotype’ [24,33].

During an examination of specimens collected between October and December 2023 from the Huanglianshan National Nature Reserve, Lvchun, Yunnan, China, we identified four *Murina* specimens. Morphologically, these specimens appear to belong to the ‘*cyclotis*-morphotype’, but analyses of molecular and morphological characteristics indicate that the specimens do not belong to any known species of genus *Murina*. We, therefore, describe these here as a new species of the genus *Murina*.

## 2. Materials and Methods

### 2.1. Sample Collection

The four specimens in this study included two adult males and two adult females, collected from the Huanglianshan National Nature Reserve, Lvchun, Yunnan, China, in October and December 2023. The voucher specimens are stored at the Kunming Natural History Museum of Zoology, Kunming Institute of Zoology, Chinese Academy of Sciences (KIZ, CAS), Kunming, China, under field collection numbers KIZ20230972, 20230973, 20231014, and 20231194 (the corresponding museum collection numbers are KIZ025484, 025485, 025486, and 025487, respectively). All novel sequences were deposited in the NCBI GenBank database under accession numbers PQ815068, PQ815069, PQ815070 and PQ815071 for Cyt *b*, and PQ818017 (KIZ20230972) and PQ818018 (KIZ20230973) for COI.

### 2.2. Measurements

External measurements were taken using a digital calliper accurate to 0.01 mm. We measured the following: head-body length (HB), from the tip of the snout to the anus; tail length (TL), from the anus to the tip of the tail; ear length (E), from the lower edge of the external auditory meatus to the tip of the pinna; hindfoot length (HF), from the extremity of the heel to the tip of the longest toe, not including any claws; tibia length (TIB), from the knee joint to the ankle; forearm length (FA), from the elbow to the carpus, with the wings folded; and the lengths of the metatarsals of the second, third, fourth and, fifth digits (MET2, MET3, MET4 and MET5, respectively), from the carpus to the end of the respective metacarpals. Body weight (WT) was measured using an electronic scale accurate to 0.1 g.

Cranial and dental measurements were taken to the nearest 0.01 mm using a digital calliper under a stereomicroscope by Xin Mou, with the definitions and standards for craniodental measurements referenced from Velazco et al. [38] and Chen et al. [25], summarised as follows: total length of skull (STOTL), from the anterior rim of the alveolus of the first upper incisor (I^2^) to the point projecting out of the occipital region the most; greatest length of skull (GTL), from the anterior aspect of I^2^ to the most prominent point of the occipital region; condylocanine length (CCL), from the exoccipital condyle to the most anterior part of C^1^; condylobasal length (CBL), from the exoccipital condyle to the posterior rim of the alveolus of I^2^; braincase width (BCW), the greatest width of the braincase; mastoid width (MAW), the greatest distance across the mastoid region; interorbital width (IOW), the smallest width of the interorbital constriction; lacrimal width (LW), the greatest width across the lacrimal tubercles at the rostral margins of the orbits; zygomatic width (ZYW), the greatest width of the skull across the zygomatic arches; braincase height (BCH), the distance from the horizontal plane to the highest point of the cranium, measured with the skull placed horizontally; upper canine width (C^1^C^1^W), the greatest width of the outer borders of C^1^; upper molar greatest width (M^3^M^3^W), the greatest width of the outer borders of the third upper molar (M^3^); upper canine–molar length (CM^3^L), from the anterior of C^1^ to the posterior of the M^3^ crown; upper canine–premolar length (CP^4^L), from the anterior of C^1^ to the posterior of P^4^ crown; mandible length (ML), from the anterior rim of the alveolus of I^2^ to the most posterior part of the condyle; coronoid process height (CPH), the shortest distance from the apex of the coronoid process to the indentation of the lower border of the ramus mandibula; the lower canine–molar length (CM_3_L), from anterior of the lower canines (C_1_) to posterior of the third lower molar (M_3_) crown.

### 2.3. Molecular Analyses

Total genomic DNA was extracted from muscle samples using the TSINGKE TSP202-50 Trelief ^®^ Hi-Pure Animal Genomic DNA Kit (Tsingke Biotech, Beijing, China), following the manufacturer’s protocols. The Cyt *b* and COI gene sequences were amplified and sequenced using the following primer pairs: Molcit-F (5′-AATGACATGAAAAATCACCGTTGT-3′, Ibáñez et al. [39]) and CytB-H (5′-CTTTTCTGGTTTACAAGACCAG-3′, Weyeneth et al. [40]) for Cyt *b*; COF: TTCTCAACCAACCACAAAGACATTGG and COR: TAGACTTCTGGGTGGCCAAAGAATCA (self-designed and optimised) for COI. Polymerase chain reaction (PCR) was conducted in a total volume of 50 μL, including template DNA (1 μL), each primer (10 pM, 2 μL), and GOLD mix (Green-TSINGKE TSE101) (45 μL). The PCR procedure consisted of 94 °C for 2 min; 5 cycles of 94 °C for 30 s, 50 °C for 40 s and 72 °C for 1 min; 35 cycles of 94 °C for 30 s, 55 °C for 40 s and 72 °C for 1 min; and a final extension at 72 °C for 10 min, followed by 4°C for renaturation. The PCR products were analysed via agarose gel electrophoresis and purified using the Trelief ^®^ DNA Gel Extraction Kit (Tsingke Biotech, Beijing). Finally, the purified samples were sequenced using an ABI 3730XL DNA Analyzer (Carlsbad, CA, USA) at Tsingke Biotech (Beijing, China). The sequencing files were checked and assembled using SeqMan in Lasergene v7.1 (DNASTAR Inc. Madison, WI, USA).

The Cyt *b* and COI sequences were compared to 25 Cyt *b* and 32 COI sequences of subfamily Murininae species downloaded from the National Center for Biotechnology Information (NCBI) GeneBank database using PhyloSuite v1.2.2 [41], with their accession numbers listed in Table 1. All Cyt *b* and COI sequences were aligned using the ClustalW algorithm [42] with default parameters in MEGA11 [43] and were truncated to 1140 bp and 657 bp, respectively. The genetic distances were calculated using the pairwise distance parameter based on the Kimura 2-parameter (K2P) model in the distance module of MEGA11 with a bootstrap procedure using 1000 replicates, employing the pairwise deletion option to remove ambiguous positions. ModelFinder [44] was used to select the best-fit model based on the Bayesian information criterion (BIC). Phylogenetic reconstruction for Cyt *b* and COI was carried out using Bayesian inference (BI) under the GTR+I+G+F model with MrBayes v3.2.6 [45] in PhyloSuite v1.2.2 [40], employing a partition model with two parallel runs and 2,000,000 generations, discarding the initial 25% of sampled data as burn-in. Maximum-likelihood phylogenies were inferred using IQ-TREE [46] under the GTR+I+G4+F model for Cyt *b* and TIM2+R4+F model for COI with 5000 ultrafast bootstraps [47].

## 3. Results

### 3.1. Systematic Description

*Murina lvchun* Xin Mou & Song Li, sp. nov.

Holotype: Field collection number KIZ20230972 and corresponding museum collection number KIZ025484, adult female, collected by Xin Mou and Song Li on 9 October 2023. The voucher specimen is stored at the Kunming Natural History Museum of Zoology, Kunming Institute of Zoology, Chinese Academy of Sciences (KIZ, CAS), Kunming, China. The mitochondrial Cyt *b* and COI nucleotide sequences were submitted to GenBank under accession number PQ815068 and PQ818017.

Type locality: Huanglianshan National Nature Reserve, Lvchun County, Yunnan Province, China (22.88° N, 102.30° E, 1724 m).

Paratype: Field collection number KIZ20230973 and corresponding museum collection number KIZ025485 (adult male), collected by Xin Mou and Song Li in Huanglianshan National Nature Reserve (22.88° N, 102.30° E, 1724 m) on 9 October 2023. The voucher specimen is stored at the Kunming Natural History Museum of Zoology, Kunming Institute of Zoology, Chinese Academy of Sciences (KIZ, CAS), Kunming, China. The mitochondrial Cyt *b* and COI nucleotide sequences were submitted to GenBank under accession number PQ815069 and PQ818018.

Etymology: The name *lvchun* denotes the toponym for the type (and only) locality of the species.

Measurements: Measurements of *Murina lvchun* are shown in Table 2.

**Figure 1:**
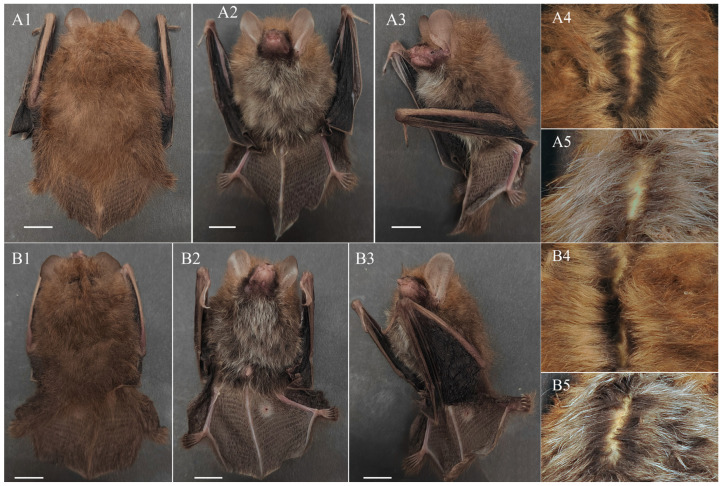
External morphology of *Murina Lvchun* Xin Mou & Song Li, sp. nov. (**A**) Holotype; (**B**) paratype. Dorsal (1), ventral (2) and lateral (3) views of unprocessed specimens; dorsal (4) and ventral (5) close-up photographs of stuffed specimens made after alcohol immersion. Scale = 10 mm.

**Figure 2:**
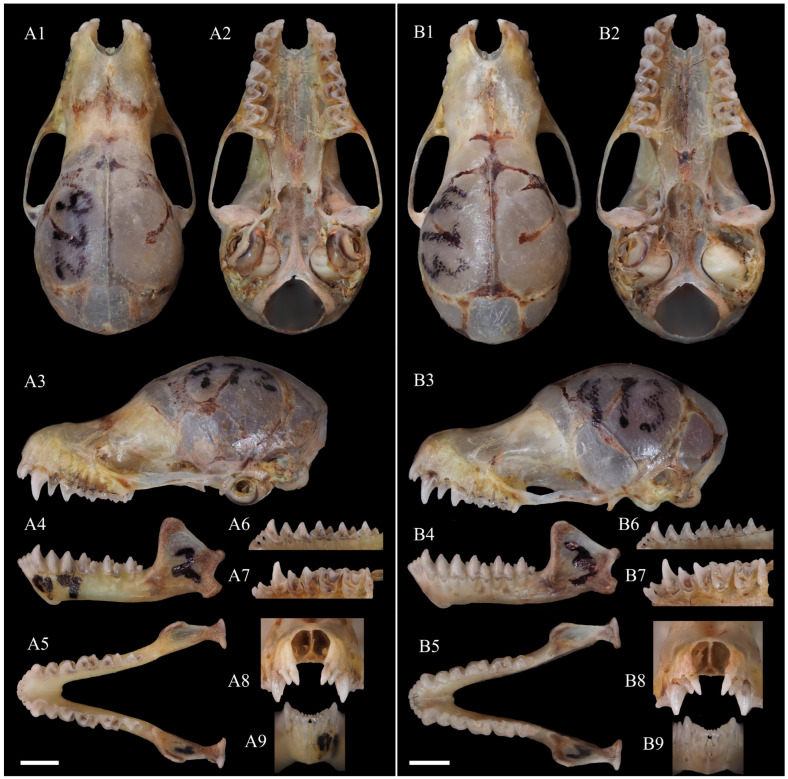
Skull of *Murina lvchun* Xin Mou & Song Li, sp. nov. (**A**) Holotype; (**B**) paratype. Dorsal (1), ventral (2) and lateral (3) views of the skull; lateral (4) and occlusal (5) views of the mandible; lingual view of the mandibular (6) and maxillary (7) dentition; labial view of the upper (8) and lower (9) incisors and canines. Scale = 2 mm.

Diagnosis: Medium-sized *Murina* species, FA 32.76–35.36 mm and GTL 16.45–16.73 mm (Table 3). Tubular nostrils, lower part of the posterior border of the ear slightly emarginate, and tragus about half the length of the ear and notched at the base. Plagiopatagium attached close to the base of the claw on the outer toe. Dorsal hairs are reddish brown overall, with tricoloured bands that are reddish brown apically, lighter in the middle and grey–black at the bases; ventral hairs are greyish-white on the thorax and abdomen and brown on the shoulders, with grey-black bases. Forearms sparsely furred, and hind feet and dorsal part of the uropatagium densely furred near the body. A ring of black stiff hairs around the snout and eyes (Figure 1). Cerebral cranium rounded, with pronounced rostral concavity; sagittal crest slightly pronounced and lambdoid crest inconspicuous. In lateral view, I^2^ is partially obscured by I^3^, P^2^ is elongated but slightly shorter than P^4^ and longer than 2/3 of C^1^, and P^4^ is wider and slightly shorter than C^1^. In the maxillary occlusal view, crown areas of C^1^ and P^4^ are approximately equal; that of P^2^ is greater than 2/3 of P^4^; mesostyle is not very well developed in M^1^ and M^2^; and M^3^ is reduced. In the mandible, heights of P_2_ and P_4_ approximately equal and about 3/4 of C_1_, the crown areas of C_1_ and P_4_ are approximately equal, and the crown area of P_2_ about is 2/3 that of C_1_ and P_4_. M_1_ and M_2_ are short and broad, and their hypoconid is acute and connected to the hypoconulid by the postcristid (Figure 2).

Description: Body: Medium-sized *Murina* species, WT 5–6.5 g, HB 41.3–46.11 mm, FA 32.76–35.36 mm, and STOTL 16.15–16.4 mm. Tubular nostrils opening sideways. Ears 14.9–16.3 mm, overall light brown, upper part almost rounded and lower part of the posterior border slightly emarginated. Tragus about half the length of the ear, slightly lighter in colour than the ear and not pointed apically, tending to widen from the tip to the base and distinctly widening about 1/3 of the way from the tip, with a distinct notch at the base. Third, fourth and fifth metacarpals are approximately equal (the third slightly longer than the fourth and fifth) and distinctly larger than the second metacarpal. HB (41.3–46.11 mm) is longer than TL (35.33–36.83 mm), tail slightly free at the tip and calcar extending in the uropatagium about 35–40% of the distance from the foot to the tip of the tail. The plagiopatagium isattached near the base of the claw on the outer toe (Figure 1 and Table 3).

Fur: Dorsal fur is denser and reddish brown overall, with the base dark grey or grey–black; the top reddish-brown; and the middle slightly lighter in colour than the top, yellowish-brown or light brown. Fur denser on the feet, the uropatagium near the body and the caudal vertebrae but sparse elsewhere; colour consistent with that of the dorsal fur. Short, sparse reddish-brown hairs on the forearms. Ventral fur greyish to greyish-brown on the thorax and abdomen, with the base dark grey or greyish-black and the top greyish to greyish-brown; light brown on the shoulders; and short, sparse and light brown on the uropatagium near the abdomen and caudal vertebrae. Shorter stiff black hairs around the muzzle and eyes, and longer, sparse greyish-white whiskers at the muzzle (Figure 1).

Skull: skull medium-sized, and GTL 16.45–16.73 mm. Sagittal crest is not well developed but clearly visible, slightly protruding from the cranial midline; the lambdoid crest is not obvious. The length of the rostrum is relatively long, with a prominent depression in the middle. In dorsal view, narial emargination is as wide as long; the braincase is almost circular; the zygomatic arch thin and straight, with almost no curvature; and the posterior edge of the skull curved. In ventral view, the palatine is narrow and slightly concave; the pterygoid is slightly curved inward; and the basisphenoid pits tear-drop-shaped, extending to the middle–lower part of the tymapanic bulla. In lateral view, the braincase is almost elliptical; the height from the snout to the parietal shows an upward trend, with a gentle slope, prominent depression between the snout and frontal end, with slight protrusion at the frontal end; and the zygomatic arch is wide and slightly robust, with the front and rear ends nearly horizontal, while the middle part slightly convex. ML 10.47–10.76 mm, and mandible slightly robust. In lateral view, the coronoid process is relatively high and no depression between it and the condyle, line connecting the condyle and angle is perpendicular to the horizontal line, with an obvious depression in the middle; and the angle is short and straight, with an obvious depression in the front (Figure 2 and Table 3).

Dentition: Dental formula, $I\frac{-2\;3}{1\;2\;3}C\frac{1}{1}PM\frac{-2-4}{-2-4}M\frac{1\;2\;3}{1\;2\;3}$ = 34. In the maxilla, I^2^ is located laterally anterior to the second incisor (I^3^) and partially visible in the lateral view; crown area of P^2^ is larger than 2/3 that of P^4^, and crown area of C^1^ is roughly equal to that of P^4^. *Murina lvchun* clearly belongs to the ‘*cyclotis*-morphotype’, based on the above characteristics. Upper tooth rows converge slightly anteriorly; C^1^C^1^W/M^3^M^3^W ratio = 0.69–0.72. I^2^ is slightly higher than I^3^, with area less than half that of I^3^; I^2^ with two cusps, a smaller secondary cusp located behind the primary cusp. C^1^, without any secondary cusps, was significantly higher than P^2^ and P^4^; P^2^ is slightly lower than P^4^; P^2^ elongated; and P^4^ wider. In occlusal view, C^1^ is not rounded, P^2^ is slightly inclined rectangular and P^4^ slightly trapezoidal. The mesostyles of M^1^ and M^2^ is slightly reduced, but the M^1^ and M^2^ surfaces are still distinctly W-shaped and concave; the paracone, metacone and protocone are well developed, with the metacone slightly higher than the paracone; the trigon basin open and the talon well developed, with the antero-external valley area significantly smaller than that of the postero-external valley. M^3^ reduced, with only parastyles, paracones, and protocones; M^1^ of holotype has a more pointed mesostyle, subequal in height to the parastyle and metastyle; the mesostyle of M^2^ relatively rounded, distinctly lower than the parastyle and metastyle; and M^3^ reduced, with part of its structure lost. In the mandible, the first, second and third lower incisors (I_1_ I_2_, and I_3_) tricuspid and the outer cusps of I_1_, I_2_ and I_3_ slightly overlapped. Height gradually increases from I_1_ to I_3_, with C_1_ significantly higher than I_3_ and slightly higher than P_2_ and P_4_, approximately equal in height. The basal areas of C_1_ and P_4_ approximately equal, the basal area of P_2_ about 2/3 that of C_1_ and P_4_. In occlusal view, the trigonid of M_1_ and M_2_ approximately equilateral triangular in shape; the talonid wider and shorter, resulting in M_1_ and M_2_ appearing shorter and wider; the protoconid of M_1_ and M_2_ relatively rounded; the hypoconid more acute, with the protoconid and hypoconid of M_3_ the opposite; and the talonid of M_3_ slightly reduced. In labial view, the height of the hypoconid of M_1_ and M_2_ about half of that of the protoconid, whereas the height of the paraconid and metaconid about 1/3 of that of the protoconid; hypoconid connected to the hypoconulid by the postcristid, a tooth shape known as the myotodont type (Figure 2).

Comparisons: Based on its dentition, *M. lvchun* can be distinguished from all members of the ‘*suilla*-morphotype’ currently described as, in the members of the ‘suilla-morphotype’, I^2^ is anterior to I^3^, I^2^ is clearly visible in the lateral view, and the crown area of P^2^ is half or less than that of P^4^. Meanwhile, within the ‘*cyclotis*-morphotype’, *M. aenea*, *M. fionae*, *M. guilleni*, *M. harrisoni*, *M. huttoni*, *M. peninsularis*, *M. puta* and *M. tiensa* were all greater than *M. lvchun* in measurements of their GTL, STOTL, CBL and ML (if any), while *M. lorelieae*, *M. rongjiangensi*, *M. rozendaali* and *M. shuipuensis* were significantly smaller than *M. lvchun* in their GTL and CBL measurements; furthermore, the FA and CBL of female and male specimens of *M. liboensis* were smaller than those of female and male specimens of *M. lvchun*, respectively (Table 4).

**Figure 3:**
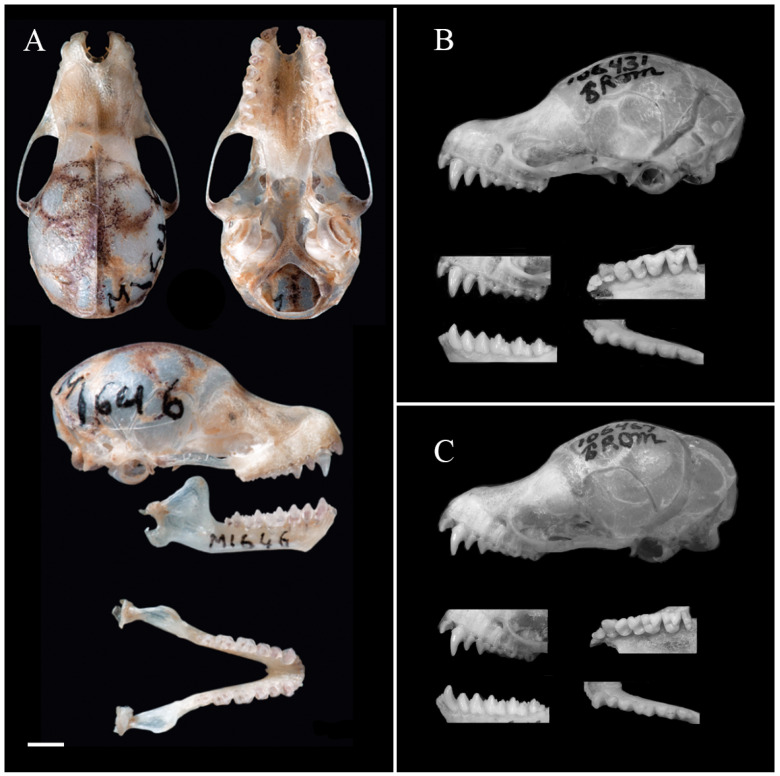
Skulls from principal species within the *Murina* genus for comparative analysis, sourced from the literature: (**A**) *M. pluvialis* (Ruedi et al. [29]); (**B**) *M. cyclotis* (Francis and Eger [30]); (**C**) *M. annamitica* (Francis and Eger [30]). Scale = 2 mm.

Comparison with *M. pluvialis*: In the ‘*cyclotis*-morphotype’, *M. lvchun* is closest to *M. pluvialis* in external morphology and craniodental measurements. Nevertheless, the two species can be distinguished by the following characteristics: The overall colouration is different; although both reddish-brown, the former is more brown and latter more red. The sagittal crest and lambdoid crest of *M. pluvialis* are distinct, whereas the sagittal crest of *M. lvchun* is distinct but not as well developed as *M. pluvialis*, and the lambdoid crest is not readily apparent. The rostral depression of *M. lvchun* is obviously deeper. In lateral view of the mandible, the condyle of *M. pluvialis* is thicker and its depression from the angle is more obvious. In lateral view, the degree of obstruction of I^2^ is different; P^2^ of *M. pluvialis* appears short, with a height less than half of C^1^ and about 2/3 of the height of P^4^, while P^2^ of *M. lvchun* appears slender, with a height greater than 2/3 of C^1^ and slightly less than the height of P^4^. In the mandible, the hypoconids of M_1_ and M_2_ of *M. lvchun* appear more pointed and the hypoconid is connected to the hypoconulid by the postcristid, while the hypoconids of M_1_ and M_2_ of *M. pluvialis* are more rounded, and Ruedi et al. [29] have described that the postcristid is connected to the entoconid (Figure 1 and Figure 3A).

Comparison with *M. cyclotis*: Although the body size and craniodental measurements of *M. cyclotis* overlap with those of *M. lvchun*, there are still stable differences that distinguish them. The overall colour of *M. lvchun* is darker, and there is a mask of black bristles around the snout and eyes, which is not present in *M. cyclotis*. The sagittal and lambdoid crests of *M. lvchun* are not as obvious as those of *M. cyclotis*. In lateral view, the shape of P^2^ and the height of P^2^ relative to P^4^ are different between the two species. In occlusal view, the shapes of P^2^ and P^4^ are different between the two species, and the sizes of P^2^ and P^4^ of *M. cyclotis* are similar, while P^2^ of *M. lvchun* is obviously smaller than P^4^; M^1^ and M^2^ of *M. cyclotis* are without mesostyles and their labial surfaces have a U-shaped indentation, while that of *M. lvchun* has mesostyles and the surface is W-shaped. In the mandible, the height of P_2_ and P_4_ of *M. cyclotis* is about 2/3 of C_1_, while that of *M. lvchun* is clearly greater than 2/3 of C_1_ (Figure 1 and Figure 3B).

Comparison with *M. annamitica*: The body size and craniodental measurements of *M. annamitica* are smaller than those of *M. lvchun.* The overall colour of the dorsal hairs of the two species varies, with *M. annamitica* being yellowish-brown to brown and *M. lvchun* being brown. *M. annamitica* lacks a mask of black bristles on the snout and around the eyes; the posterior margin of the ear is not concave in *M. annamitica* but is slightly concave in *M. lvchun*. The maxillary tooth rows of *M. annamitica* are almost parallel to each other, whereas those of *M. lvchun* converge slightly anteriorly. In lateral view, the extent to which I^2^ is obscured; the relative heights of P^2^, P^4^ and C^1^; as well as the heights of mandibles P_2_ and P_4_ in relation to C_1_ of the two species are different. In occlusal view, the shape of P_2_ and the ratio of the talonid to trigonid area in M_1_ and M_2_ are different (Figure 1 and Figure 3C).

**Figure 4:**
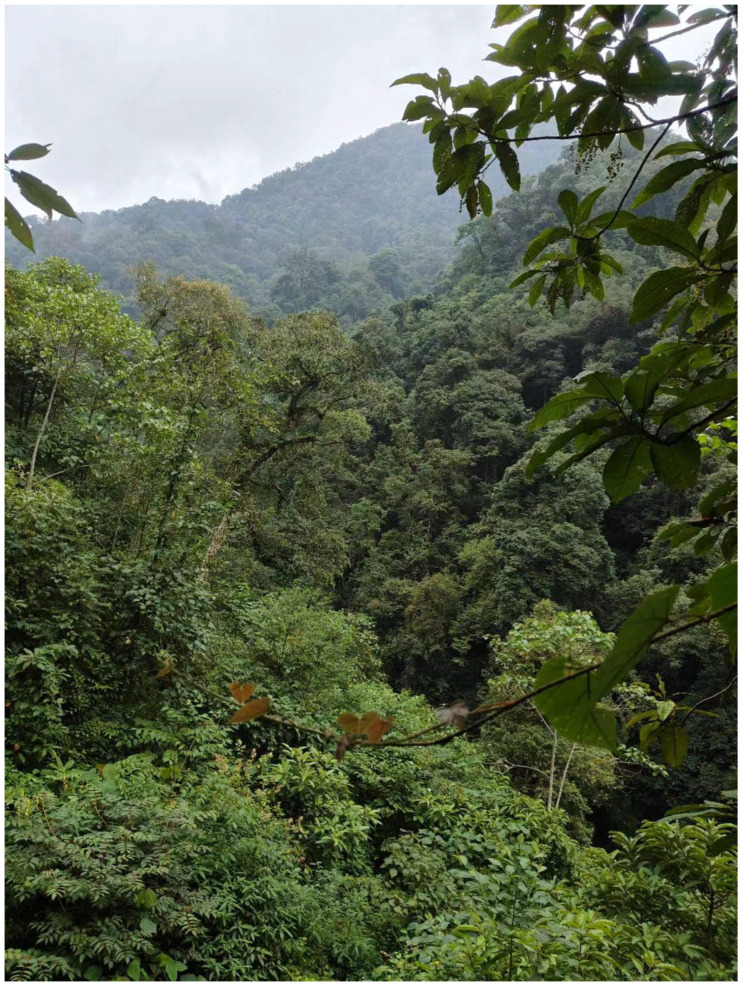
Habitat at type locality of *Murina lvchun* Xin Mou & Song Li, sp. nov. at Huanglianshan National Nature Reserve, lvchun, Yunnan, China.

Distribution and habitat: Specimens of *M. lvchun* were collected from two different locations near the stream downstream from the Huanglianshan Reservoir in the Yakou Conservation Office of the Huanglianshan National Nature Reserve in Yunnan (22. 88° N, 102. 30° E, 1724 m and 22. 89° N, 102. 31° E, 1825 m; dominated by wet monsoon evergreen broad-leaved forest, Figure 4). The high humidity, the dense forest in the area, and the absence of caves and houses in the vicinity suggest that *M. lvchun* may be an arboreal bat.

### 3.2. Molecular Phylogenetic Analyses

**Figure 5:**
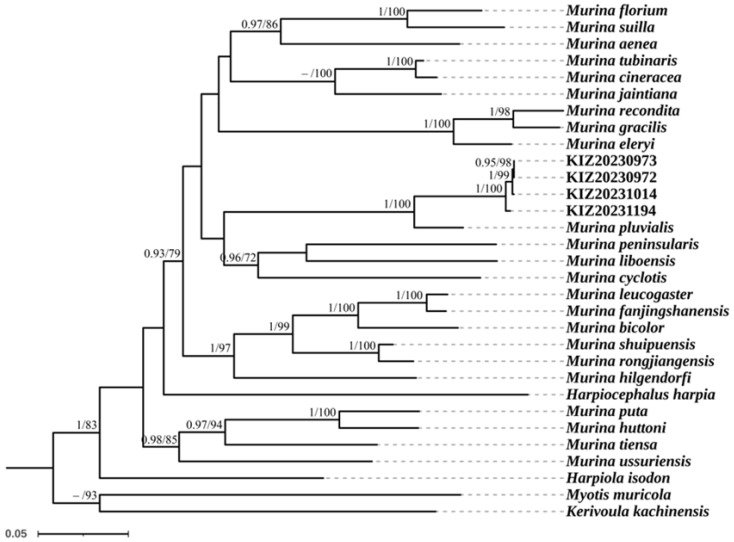
Maximum-likelihood phylogenetic tree of *Murina* species based on Cyt *b* fragments. Node numbers before “/” indicate Bayesian posterior probabilities (values below 0.90 not shown), and numbers after “/” indicate ultrafast bootstrap support in maximum-likelihood analyses (values below 70 not shown).

**Figure 6:**
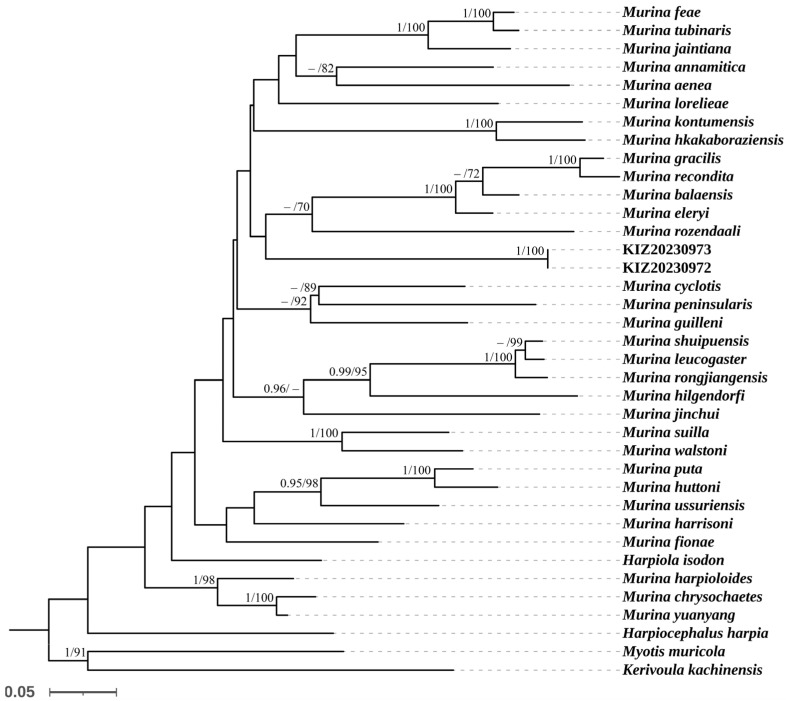
Maximum-likelihood phylogenetic tree of *Murina* species based on COI fragments. Node numbers before “/” indicate Bayesian posterior probabilities (values below 0.90 not shown), and numbers after “/” indicate ultrafast bootstrap support in maximum-likelihood analyses (values below 70 not shown).

Bayesian inference and maximum-likelihood phylogenies resulted in similar topologies: The reconstructed phylogenetic tree based on the Cyt *b* gene sequences revealed that all specimens of *M. lvchun* formed a clade and a distinct lineage sister to *M. pluvialis* with a posterior probability of 1 and an ultrafast bootstrap value of 100. The reconstructed phylogenetic tree based on the COI gene sequences revealed that two specimens of *M. lvchun* (the COI gene sequences of KIZ20231014 and KIZ20231194 were not sequenced) clustered alone in a clade but did not form a distinct lineage sister to other species (Figure 5 and Figure 6). The smallest genetic distances between *M. lvchun* and *Murina* species were found for *M. pluvialis* (7.2–7.4% in the Cyt *b* gene sequences). In addition, the genetic distances between *M. lvchun* and other species of *Murina* were greater than 12% for both the Cyt *b* gene and COI gene sequences (Table 5 and Table 6).

## 4. Discussion

We conducted an in-depth analysis of four specimens of *M. lvchun* using morphological and molecular approaches: morphologically, *M. lvchun* belong to the ‘*cyclotis*-morphotype’; can be distinguished from all members of the ‘*suilla*-morphotype’ and can be distinguished from the ‘*cyclotis*-morphotype’ *M. aenea*, *M. huttoni*, *M. harrisoni*, *M. fionae*, *M. peninsularis*, *M. puta*, *M. tiensa*, *M. guilleni*, *M. rozendaali*, *M. liboensis*, *M. lorelieae*, *M. shuipuensis,* and *M. rongjiangensis* according to body and skull size. We compared *M. lvchun* with *M. pluvialis*, *M. cyclotis,* and *M. annamitica* in detail (whose measurements are close to *M. lvchun*, among which *M. pluvialis* is most similar to *M. lvchun*) and found that these three species have discrete differences in morphology and skull parameters with respect to *M. lvchun*. At the molecular level, we sequenced the Cyt *b* and COI genes of *M. lvchun*. In the phylogenetic tree constructed based on the Cyt *b* gene sequence, *M. lvchun* and *M. pluvialis* formed a sister clade with a posterior probability of 1 and an ultrafast bootstrap value of 100; in the phylogenetic tree constructed based on the COI gene sequence, as there were no COI sequences of *M. pluvialis*, *M. lvchun* formed an independent branch and did not form a sister branch with any other species. This suggests that, among the 37 *Murina* species with Cyt *b* or COI gene sequences in Genebank (including all ‘*cyclotis*-morphotype’ members), *M. lvchun* and *M. pluvialis* are molecular sister taxa. At the same time, the interspecific genetic distance between the two species in the Cyt *b* gene sequence was 7.2–7.4%. Genetic distances less than 2% usually indicate intraspecific variation [51,52,53], and Hebert et al. [54] have suggested that the standard threshold for species differentiation should be 10 times the average genetic distance within the species; Baker and Bradley [55] stated that a genetic distance of 5% usually indicates a high probability of the existence of unrecognised genetic species. Although the above thresholds are subjective values selected from a review of published mammalian genetic distances, they are still of great importance for taxonomic research. The genetic distance between *M. lvchun* and *M. pluvialis* was greater than 5% and was more than 10 times the average genetic distance within *M. lvchun* (0.42%). This suggests that *M. lvchun* is probably a new species, genetically independent of *M. pluvialis*. In terms of morphology, *M. lvchun* can be distinguished from *M. pluvialis* based on their fur colour; their sagittal and lambdoid crests; their rostral depression; the thickness of their condyle; the degree of depression between their condyle and the angle; the relative heights of their P^2^, C^1^ and P^4^; the type of dentition of their M_1_ and M_2_ (*M. lvchun* is of the myotodont type, where the hypoconid is linked to the hypoconulid by the postcristid; *M. pluvialis* is of the nyctalodont type, where the entoconid and hypoconid are linked by the postcristid); and the shape of their hypoconid. These characteristic differences are sufficient to support the assertion of *M. lvchun* as a new species independent of *M. pluvialis*. In addition, the habitats of the two species are also quite different. According to the description of Ruedi et al. [29], the geographical coordinates of *M. pluvialis* are N 25°13′, E 91°40′, 780 m above sea level, and the habitat is secondary, dense evergreen forest, with the specimens collected in a small bamboo forest mixed with other plants. Meanwhile, the locality of *M. lvchun* belongs to wet monsoon evergreen broad-leaved forest (22. 88° N, 102. 30° E, 1724 m and 22. 89° N, 102. 31° E, 1825 m), which is more than 1000 m higher than the altitude recorded for *M. pluvialis*, and there is no obvious bamboo forest around the capture site. We, therefore, concluded that *M. lvchun* is a new species of *Murina*.

Although mtDNA-based barcoding sometimes performs poorly in taxonomic identification [56] (blurred species boundaries), manifested in overestimation of the number of putative species [57], missing taxa due to mtDNA introgression [58,59], or the suggestion of phantom lineages [60], an mtDNA analysis is currently the cheapest method to understand the genetic structure of unidentified species and the associated data are relatively complete (with more species sequences available for comparison). Therefore, it is still an important molecular marker to indicate whether a population has undescribed taxa. The genetic distance based on Cyt *b* between *M. lvchun* and *M. pluvialis* was 7.2–7.4%, which is greater than 5%, suggesting that *M. lvchun* is likely to be an undescribed new species. This inference was further corroborated by the results of a morphological study, indicating a strong congruence between molecular and morphological evidence in supporting the distinct taxonomic status of *M. lvchun.*

Due to the low migratory activity, high flight manoeuvrability and relatively low natural population density of *Murina*, the species composition of the genus may still be underestimated, despite the fact that its membership has more than doubled over the last two decades. The discovery of *Murina lvchun* Xin Mou & Song Li, sp. nov. has resulted in an increased number of known species in the genus *Murina*.

## 5. Conclusions

A new ‘*cyclotis*-morphotype’ species of *Murina*, *Murina lvchun* Xin Mou & Song Li, sp. nov., was described based on four specimens collected from the Huanglianshan National Nature Reserve, Lvchun, Yunnan, China. At present, the new species is known only from its type locality. Due to the presence of the Huanglianshan National Nature Reserve, there is relatively less human activity in the type locality. Therefore, the local ecological environment is relatively well preserved, and this species is apparently not threatened.

## Figures and Tables

**Table 1:** Species and GenBank accession numbers of sequences used for phylogenetic reconstruction.

Taxon	Accession Number		Taxon	Accession Number	
	COI	Cyt *b*		COI	Cyt *b*
*H. harpia*	HM540274	MH137366	*M. huttoni*	JQ601452	KU521385
*H. isodon*	HM540286	GQ168914	*M. jaintiana*	MF537346	JQ044690
*K. kachinensis*	MZ438743	MH137392	*M. jinchui*	MN549070	–
*M. aenea*	HM540928	GQ168906	*M. kontumensis*	KT820760	–
*M. annamitica*	HM540969	–	*M. leucogaster*	HM540988	GQ168912
*M. balaensis*	KY034093	–	*M. liboensis*	–	MK095626
*M. bicolor*	–	JQ044696	*M. lorelieae*	JN082179	–
*M. chrysochaetes*	HM540986	–	*M. peninsularis*	HM540972	GQ168911
*M. cineracea*	–	JQ044693	*M. pluvialis*	–	JQ044689
*M. cyclotis*	JF443973	MG194466	*M. puta*	KT982277	GQ168901
*M. eleryi*	KT762293	GQ168908	*M. recondita*	KJ198687	KJ198269
*M. fanjingshanensis*	–	KT180333	*M. rongjiangensis*	MN549085	MF359930
*M. feae*	KY034072	–	*M. rozendaali*	KY034110	–
*M. fionae*	HM540965	–	*M. shuipuensis*	JN082180	MK747249
*M. florium*	–	GQ168902	*M. suilla*	KY034090	GQ168905
*M. gracilis*	KJ198567	KJ198141	*M. tiensa*	–	GQ168922
*M. guilleni*	KY034137	–	*M. tubinaris*	HM541000	GQ168904
*M. harpioloides*	JF443974	–	*M. ussuriensis*	HQ974648	JX872285
*M. harrisoni*	MN549045	–	*M. walstoni*	HM540957	–
*M. hilgendorfi*	JF442833	MG897567	*M. yuanyang*	PQ179689	–
*M. hkakaboraziensis*	MF537343	–	*M. muricola*	MW054913	MH137365

**Table 2:** Weight, external, and craniodental measurements of the *Murina lvchun* Xin Mou & Song Li, sp. nov. “♂” indicates male; “♀” indicates female.

Character	KIZ20230972 Holotype, ♀	KIZ20230973 Paratype, ♂	KIZ20231014 ♀	KIZ202301194 ♂
WT	5.8	5	6.4	5.6
HB	44.34	41.30	46.11	42.74
TL	35.74	35.74	36.83	35.33
E	14.90	16.34	16.21	15.84
HF	8.11	8.21	8.27	8.02
TIB	17.80	16.90	17.73	17.39
FA	34.34	32.76	35.36	33.41
MET2	27.88	26.92	28.39	26.59
MET3	31.71	30.29	31.98	31.16
MET4	31.33	30.2	31.15	30.33
MET5	31.68	29.08	31.49	30.56
STOTL	16.16	16.15	16.40	16.18
GTL	16.52	16.45	16.73	16.58
CCL	14.54	14.36	14.84	14.44
CBL	15.21	14.96	15.32	15.28
BCW	7.82	7.68	8.17	7.96
MAW	7.69	7.59	7.77	7.76
IOW	4.29	4.28	4.39	4.44
LW	4.88	4.91	5.08	4.93
ZYW	9.05	8.82	9.58	9.20
BCH	7.79	7.60	7.71	7.71
C^1^C^1^W	4.14	3.95	4.08	3.89
M^3^M^3^W	5.74	5.60	5.95	5.64
CM^3^L	5.50	5.38	5.67	5.36
CP^4^L	2.30	2.42	2.56	2.34
ML	10.7	10.51	10.76	10.47
CPH	4.01	3.74	4.08	3.76
CM_3_L	5.95	5.78	6.00	5.82

**Table 3:** Weight (g), external and craniodental measurements (in mm) of some *Murina* species. “♂” indicates male; “♀” indicates female. Sample sizes that differ from the total number of specimens are given in italics. Data of *M. pluvialis*, *M. annamitica* and *M. cyclotis* were taken from the literature.

	*M. lvchun* Xin Mou & Song Li, sp. nov.			*M. pluvialis* ♀ Ruedi et al. [29]	*M. annamitica* Francis and Eger [30]		*M. cyclotis* Soisook et al. [23]	
Characteristic	Mean ± SD, min–max (*n* = 4)	♀ (*n* = 2) Means, min–max	♂ (*n* = 2), Means, min–max		♂ (*n* = 5), Means, min–max	♀	♀ (*n* = 40), Means ± SD, min–max	♂ (*n* = 36), Means ± SD, min–max
WT	5.7 ± 0.50, 5.0–6.4	6.1, 5.8–6.4	5.3, 5.0–5.6	–	5.1, 4.7–5.3,	9.6	–, 6.6, *1*	–, 5.0–5.8, *2*
HB	43.62 ± 1.79, 41.30–46.11	45.23, 44.34–46.11	42.02, 41.30–42.74	–	–	–	45.1 ± 2.8, 41.1–50.0, *17*	42.3 ± 2.3, 38.7–46.4, *16*
TL	35.91 ± 0.56, 35.33–36.83	36.29, 35.74–36.83	35.54, 35.33–35.74	–	–	–	37.4 ± 2.5, 32.0–41.1, *17*	34.9 ± 3.9, 26.2–39.0, *17*
E	15.82 ± 0.56, 14.90–16.34	15.56, 14.90–16.21	16.09, 15.84–16.34	–	13.4, 13.1–14.0, *4*	14.5	14.5 ± 1.0, 12.7–16.0, *17*	14.0 ± 1.3, 12.0–17.6, *17*
HF	8.15 ± 0.10, 8.02–8.27	8.19, 8.11–8.27	8.12, 8.02–8.21	–	–	–	8.3 ± 0.8, 7.0–9.7, *17*	7.9 ± 0.6, 6.5–8.8, *17*
TIB	17.46 ± 0.36, 16.90–17.80	17.77, 17.73–17.80	17.15, 16.90–17.39	–	–	–	18.8 ± 0.9, 17.3–20.3	17.6 ± 1.1, 14.5–19.3, *18*
FA	33.97 ± 0.98, 32.76–35.36	34.85, 34.34–35.36	33.09, 32.76–33.41	36.60	30.6, 30.0–30.9	32.2	33.9 ± 1.0, 31.6–36.8	30.7 ± 0.9, 29.4–33.0, *35*
MET2	27.45 ± 0.72, 26.59–28.39	28.14, 27.88–28.39	26.76, 26.59–26.92	–	–	–	–	–
MET3	31.29 ± 0.65, 30.29–31.98	31.85, 31.71–31.98	30.73, 30.29–31.16	–	–	–	32.0 ± 1.2, 30.4–34.9, *11*	29.2 ± 1.5, 26.5–32.4, *16*
MET4	30.75 ± 0.49, 30.20–31.33	31.24, 31.15–31.33	30.27, 30.20–30.33	–	–	–	31.4 ± 1.3, 29.7–34.4, *11*	28.3 ± 1.7, 24.7–31.9, *16*
MET5	30.70 ± 1.03, 29.08–31.68	31.59, 31.49–31.68	29.82, 29.08–30.56	–	–	–	32.3 ± 1.2, 30.8–35.1, *11*	29.0 ± 1.6, 25.1–31.8, *16*
STOTL	16.22 ± 0.10, 16.15–16.40	16.28, 16.16–16.40	16.17, 16.15–16.18	16.40	–	–	–	–
GTL	16.57 ± 0.10, 16.45–16.73	16.63, 16.52–16.73	16.52, 16.45–16.58	–	16.1, 16–16.2	16.4	17.21 ± 0.47, 16.60–18.18, *25*	16.47 ± 0.34, 15.86–17.08, *27*
CCL	14.55 ± 0.18, 14.36–14.84	14.69, 14.54–14.84	14.40, 14.36–14.44	14.50	–	–	15.22 ± 0.41, 14.34–16.17	14.45 ± 0.34, 13.6–15.12
CBL	15.19 ± 0.14, 14.96–15.32	15.27, 15.21–15.32	15.12, 14.96–15.28	–	14.6, 14.3–14.8	15.1	15.85 ± 0.48, 14.95–16.86, *24*	14.97 ± 0.4, 14–15.67, *27*
BCW	7.91 ± 0.18, 7.68–8.17	8.00, 7.82–8.17	7.82, 7.68–7.96	–	–	–	7.71 ± 0.19, 7.4–8.17, *38*	7.64 ± 0.22, 7.16–8.10
MAW	7.70 ± 0.07, 7.59–7.77	7.73, 7.69–7.77	7.68, 7.59–7.76	7.78	7.8, 7.5–8	8.1	8.20 ± 0.21, 7.64–8.58, *38*	7.9 ± 0.26, 7.11–8.48
IOW	4.35 ± 0.07, 4.28–4.44	4.34, 4.29–4.39	4.36, 4.28–4.44	4.32	–	–	4.25 ± 0.13, 3.99–4.52, *38*	4.17 ± 0.12, 3.92–4.48
LW	4.95 ± 0.08, 4.88–5.08	4.98, 4.88–5.08	4.92, 4.91–4.93	–	–	–	–, 4.67–5.85, *23*	4.95 ± 0.24, 4.26–5.42, *27*
ZYW	9.16 ± 0.28, 8.82–9.58	9.32, 9.05–9.58	9.01, 8.82–9.20	9.26	8.8, 8.7–9	9.4	9.84 ± 0.29, 9.33–10.43, *37*	9.36 ± 0.31, 8.78–10.05, *35*
BCH	7.70 ± 0.07, 7.6–7.79	7.75, 7.71–7.79	7.66, 7.60–7.71	6.43	–	–	6.50 ± 0.24, 6.10–7.21, *37*	6.49 ± 0.3, 6.08–7.22, *34*
C^1^C^1^W	4.02 ± 0.10, 3.89–4.14	4.11, 4.08–4.14	3.92, 3.89–3.95	4.21	4, 3.8–4.1	4.5	4.25 ± 0.14, 4.00–4.68, 37	4.00 ± 0.14, 3.73–4.27
M^3^M^3^W	5.73 ± 0.14, 5.6–5.95	5.85, 5.74–5.95	5.62, 5.60–5.64	5.52	5.3, 5–5.4	5.5	5.57 ± 0.19, 5.18–6.05, *38*	5.39 ± 0.18, 5.07–5.79, *35*
CM^3^L	5.48 ± 0.12, 5.36–5.67	5.59, 5.50–5.67	5.37, 5.36–5.38	5.49	5.1, 5–5.2	5.3	5.61 ± 0.19, 5.06–6.00, *38*	5.41 ± 0.15, 5.12–5.68
CP^4^L	2.41 ± 0.10, 2.30–2.56	2.43, 2.30–2.56	2.38, 2.34–2.42	–	–	–	2.76 ± 0.19, 2.21–3.11, *38*	2.66 ± 0.16, 2.21–2.96
ML	10.61 ± 0.12, 10.47–10.76	10.73, 10.70–10.76	10.49, 10.47–10.51	11.18	–	–	11.86 ± 0.35, 11.32–12.78, *37*	11.17 ± 0.3, 10.52–11.68, *35*
CPH	3.90 ± 0.15, 3.74–4.08	4.045, 4.01–4.08	3.75, 3.74–3.76	4.02	–	–	4.71 ± 0.27, 4.16–5.30, *38*	4.14 ± 0.21, 3.77–4.60, *35*
CM_3_L	5.89 ± 0.09, 5.78–6.00	5.98, 5.95–6.00	5.80, 5.78–5.82	5.93	5.5, 5–5.7	5.9	6.11 ± 0.16, 5.75–6.49, *37*	5.84 ± 0.14, 5.57–6.18

**Table 4:** Body and skull size of species in ‘*cyclotis*-morphotype’. “♂” indicates male; “♀” indicates female. Sample sizes that differ from the total number of specimens are given in italics. Data for other species were taken from the literature.

Taxon	Sex	n	FA	GTL	STOTL	CBL	ML
*M. lvchun* Xin Mou & Song Li, sp. nov.	♀	2	34.85, 34.34–35.36	16.63, 16.52–16.73	16.28, 16.16–16.40	15.27, 15.21–15.32	10.73, 10.70–10.76
	♂	2	33.09, 32.76–33.41	16.52, 16.45–16.58	16.17, 16.15–16.18	15.12, 14.96–15.28	10.49, 10.47–10.51
*M.aenea*, Bumrungsri et al. [48]	♀	2	35.2, 34.7–35.7	17.5, 17.2–17.8	–	15.9, 15.8–16.0	12.4, 11.9–12.7
*M. fionae*, Soisook et al. [23]	♀	6	37.3, 35.5–40.1	18.80, 18.12–19.19	–	17.06, 16.48–17.45	12.82, 12.56–13.01
	♂	7	35.1, 34.5–36.3	18.54, 17.53–19.26	–	16.80, 15.99–17.49	12.48, 11.99–13.19
*M. guilleni*, Soisook et al. [23]	♀	3	35.4, 35.0–35.9	17.65, 17.12–18.10	–	16.10, 15.79–16.43	12.11, 11.95–12.34
	♂	6	33.2, 31.9–34.0	17.10, 16.40–17.54	–	15.46, 14.93–15.83	11.38, 11.13–11.74
*M. harrisoni*, Csorba and Bates [17]	♀	1	35.9	–	18.39	–	13.03
*M. huttoni*, Son et al. [49]	♀	3	–	–	18.01, 17.85–18.10	–	12.19, 12.15–12.25
	♂	4	–	–	17.45, 16.71–17.85	–	11.85–11.95
*M. liboensis*, Zeng et al. [26]	♀	2	32.46, 32.28–32.64	16.95, 16.92–16.97	–	13.60, 13.35–13.85	11.96, 11.63–12.28
	♂	3	28.16–30.98	15.54–16.52	–	10.16–11.54	9.67–11.53
*M. lorelieae*, Eger and Lim [28]	♂	1	30.8	15.52	–	14.11	9.6
*M. peninsularis*, Soisook et al. [22]	♀	19	37.7, 34.5–39.4	18.70, 17.59–19.33, *13*	–	17.11, 16.11–17.69, *13*	12.75, 12.09–13.59
	♂	23	35.7, 33.8–38.1, *22*	17.79, 17.39–18.52, *17*	–	16.06, 15.68–16.91, *17*	11.92, 11.25–12.92, *21*
*M. puta*, Csorba and Bates [17]	–	–	30–37	–	16.63–18.09, *20*	–	11.43–12.45, *17*
*M.rongjiangensis*, Chen et al. [25]	♀	3	29.97–34.21	15.75–16.38	–	12.49–13.26	10.37–11.24
	♂	1	30.4	16.18	–	12.95	10.68
*M. rozendaali*, Hill and Francis [50]	♀	2	32.7, 32.3–33.1	15.8, 15.6–16.0	–	14.5, 14.4–14.6	10.5, 10.4–10.5
	♂	1	32.1	15.7	–	14.4	10.5
*M. shuipuensis*, Eger and Lim [28]	♂	1	30.55	15.9	–	14.73	10.3
*M. tiensa*, Csorba et al. [18]	–	4	35.2–40.1	–	17.39–19.43	–	11.95–13.62

**Table 5:** Genetic distances (%) between species of *Murina* calculated from 1140 bp Cyt *b* gene fragment. Taxa arranged in ascending order according to the genetic distances with *Murina lvchun* Xin Mou & Song Li, sp. nov. Taxa with genetic distances greater than 17.5% from *Murina lvchun* Xin Mou & Song Li, sp. nov. not shown.

NO.	Taxon	1	2	3	4	5	6	7	8	9	10	11	12
1	KIZ20230972												
2	KIZ20230973	0.0											
3	KIZ20231014	0.2	0.2										
4	KIZ20231194	0.7	0.7	0.7									
5	*M. pluvialis*	7.4	7.4	7.4	7.2								
6	*M. suilla*	16.3	16.4	16.5	16.4	17.6							
7	*M. shuipuensis*	16.7	16.7	16.7	16.6	16.8	17.3						
8	*M. tiensa*	17.1	17.2	17.3	17.3	17.2	19.1	17.1					
9	*M. aenea*	17.3	17.5	17.6	17.5	17.7	14.5	19.1	17.8				
10	*M. liboensis*	17.4	17.3	17.1	17.3	17.2	17.2	17.7	16.8	17.7			
11	*M. florium*	17.4	17.5	17.7	17.8	18.6	8.3	16.6	18.3	15.7	18.1		
12	*M. tubinaris*	17.5	17.6	17.5	17.1	17.6	17.2	15.3	17.1	16.2	16.6	15.4	

**Table 6:** Genetic distances (%) between species calculated from 657 bp COI gene fragment. Taxa arranged in ascending order according to the genetic distances with *Murina lvchun* Xin Mou & Song Li, sp. nov. Taxa with genetic distances greater than 19.0% from *Murina lvchun* Xin Mou & Song Li, sp. nov. not shown.

NO.	Taxon	1	2	3	4	5	6	7	8	9	10	11	12
1	KIZ20230972												
2	KIZ20230973	0.0											
3	*M. chrysochaetes*	17.5	17.5										
4	*M. harpioloides*	17.7	17.7	10.3									
5	*M. puta*	17.7	17.7	16.5	18.3								
6	*M. yuanyang*	18.2	18.2	2.8	9.8	16.9							
7	*M. ussuriensis*	18.5	18.5	19.9	18.3	12.9	19.6						
8	*M. balaensis*	18.5	18.5	19.4	18.2	18.6	18.6	18.7					
9	*M. suilla*	18.7	18.7	18.4	17.2	20.1	18.5	19.8	18.1				
10	*M. huttoni*	18.8	18.8	16.6	18.4	6.7	16.2	13.9	19.6	19.2			
11	*M. hkakaboraziensis*	19.0	19.0	19.6	20.2	21.2	19.8	23.2	22.1	20.3	21.6		
12	*M. feae*	19.0	19.0	19.3	22.0	19.0	20.2	19.7	20.0	19.5	21.2	21.3	

## Data Availability

All data are presented in this article.
